# The Correlation between the Triglyceride-Glucose Index and Coagulation Markers in Patients with Recent Acute Myocardial Infarction

**DOI:** 10.1155/2022/6206802

**Published:** 2022-10-20

**Authors:** Daniel Košuta, Marko Novaković, Mojca Božič Mijovski, Borut Jug

**Affiliations:** ^1^Faculty of Medicine, University of Ljubljana, Ljubljana, Slovenia; ^2^Department of Vascular Diseases, University Medical Centre Ljubljana, Ljubljana, Slovenia; ^3^Faculty of Pharmacy, University of Ljubljana, Ljubljana, Slovenia

## Abstract

**Background:**

Metabolic abnormalities and hypercoagulability seem to have an important predictive role in patients with coronary artery disease (CAD). The triglyceride-glucose (TyG) index has emerged as a good marker for metabolic syndrome with predictive value for cardiovascular events. Overall haemostatic potential (OHP) is a reliable global haemostatic essay to identify hypercoagulability in CAD patients. The aim of our study was therefore to evaluate a possible correlation between the TyG index and haemostatic derangements in patients with CAD.

**Methods:**

Consecutive patients referred for the first follow-up visit after acute myocardial infarction between December 1, 2018, and March 31, 2020, and did not meet exclusion criteria were included. We determined OHP, overall coagulation potential (OCP), overall fibrinolytic potential (OFP), fibrinogen, D-dimer, and von Willebrand factor from peripheral blood samples. The TyG index was calculated with the previously described and validated formula. Linear regression models were constructed for the multivariate analysis.

**Results:**

A total of 117 patients (mean age 56 ± 10 years, 20% women) were included. A correlation was found between TyG index and OCP (*r* = 0.229, *p* = 0.026), TyG index and OHP (*r* = 0.202, *p* = 0.050), and TyG index and fibrinogen (*r* = 0.271, *p* = 0.005). In the multivariate model which accounted for sex, age, and BMI, the correlation between TyG index and OCP (*R*^2^ 0.108; ANOVA for regression *p* = 0.035; beta 2.08 [0.79-4.01], *p* = 0.042) and between TyG index and fibrinogen (*R*^2^ 0.11; ANOVA for regression *p* = 0.015; beta 0.35 [0.08-0.62], *p* = 0.012) emerged as statistically significant.

**Conclusion:**

The TyG index, a marker of metabolic syndrome, has a strong correlation with a hypercoagulability state in CAD, as determined by the OCP and higher fibrinogen levels. Our findings suggest that metabolic syndrome may be an important driver of atherothrombotic risk in patients with CAD.

## 1. Introduction

Coronary artery disease (CAD) is a form of atherosclerotic vascular disease associated with significant morbidity and mortality despite mounting efforts of prevention, early diagnosis, and aggressive management [1]. CAD is characterized by progressive atherosclerotic plaque build-up, which is prone to rupture, and atherothrombotic coronary artery occlusion, yielding myocardial infarction. On the one hand, atherosclerosis progression is associated with cardiovascular risk factors. While some risk factors, such as smoking, have been stagnating or declining in western populations, others—especially cardiometabolic risk factors, such as obesity, diabetes, and dyslipidaemia—have been on the rise [2, 3]. On the other hand, atherothrombotic events are associated with a procoagulant state [4], which may discern stable chronic CAD from unstable acute coronary syndromes.

Obesity and insulin resistance are notable hallmarks of cardiometabolic risk, suggesting underlying derangements in glucose and lipid metabolism. However, recent studies suggest that cardiometabolic abnormalities can be detected in nonobese individuals as well [5, 6], and identification of cardiometabolic risk beyond apparent clinical characteristics—possibly through biomarkers—is being pursued. One such biomarker is the triglyceride-glucose (TyG) index, which has been proposed as a marker of metabolic syndrome and has been associated with carotid atherosclerosis, coronary artery calcification, and higher risk of cardiovascular disease [7–9]. Moreover, recent studies have identified the TyG index as an independent predictor of coronary calcification progression, of clinical outcomes in patients with stable CAD and premature CAD, and of cardiovascular outcomes in acute coronary syndromes [7, 10–13].

In addition to risk factors-driven coronary atherosclerosis progression, haemostatic derangements play an important role in the development of myocardial infarction. CAD has been associated with a procoagulant state with mounting evidence of high haemostatic factor activity—such as increased fibrinogen levels, a procoagulant platelet response, and thrombogenicity [4, 14–17]. Procoagulant activity is associated with both the severity of chronic CAD and the likelihood of its progression to myocardial infarction [4, 18]. Since coagulation is a complex and multifactorial process, global haemostatic essays—as opposed to the measurement of individual procoagulant and fibrinolytic factors—have recently gained popularity [19]. Overall haemostatic potential (OHP) is a global haemostasis essay [20, 21], which has been validated in healthy individual as well as various groups of patients [22–24]. In CAD patients, OHP detected a hypercoagulability state, driven primarily by impaired fibrinolysis [23, 25].

Clinical features of the metabolic syndrome are associated with a prothrombotic state. Several coagulation markers are increased, and fibrinolysis is impaired in patients with metabolic syndrome, which may contribute to the adverse atherothrombotic pathophysiology and high-risk features of CAD in patients with dysmetabolic profiles [26, 27]. In the present study, we sought to appraise a possible association between the TyG index, a marker of the metabolic syndrome beyond traditional clinical features, and the OHP, an essay for haemostatic derangements, in patients with CAD.

## 2. Methods

### 2.1. Study Design and Population

Consecutive patients referred for the first follow-up visit after acute myocardial infarction between December 1, 2018, and March 31, 2019, were screened for inclusion. Inclusion criteria were 18–70 years of age and recent myocardial infarction (within 90 days from inclusion). Patients with advanced heart failure (New York Heart Association classes III and IV), patients on anticoagulation treatment, and pregnant women were excluded. The study was conducted in accordance with the Declaration of Helsinki (1964) and approved by the National Medical Ethics Committee (approval number 0120-77/2020/3).

After signing informed consent, patients were included in the study and had a fasting (>12 hours after the last meal) blood sample drawn from the cubital vein; blood was collected into 4.5 mL 0.109 M sodium citrate tubes. Citrated blood was then centrifuged for 20 minutes at 2000 × g; the extracted plasma was then stored at –70°C for further analysis.

We collected baseline data, cardiovascular risk factors, and laboratory tests (glucose (mg/dL), total cholesterol (mg/dL), HDL cholesterol (mg/dL), LDL cholesterol (mg/dL), triglycerides (mg/dL), fibrinogen (g/L), D-dimer (*μ*g/L), and von Willebrand factor (%)). The researcher who collected the aforementioned data was blinded to the coagulation test results.

Overall haemostatic potential (OHP, abs-sum), overall coagulation potential (OCP, abs-sum), and overall fibrinolytic potential (OFP, %) were determined as previously described [20, 23] using bovine thrombin (Sigma Chemical Company, St. Louis, USA) and recombinant tissue-type plasminogen activator (Actilyse 0.1 mL/mL, Boehringer Ingelheim, Germany) by absorbance measurements at 405 nm in 1-minute intervals for 40 minutes. Areas under the curve were constructed for OHP and OCP with the obtained measurements; OFP was calculated as the difference between the two aforementioned areas: OFP = [(OHP–OCP)/OCP] × 100 (%).

The TyG index was calculated with the previously described and validated formula: TyG index = ln [triglycerides (mg/dL) × plasma glucose (mg/dL)/2] [11].

### 2.2. Statistical Analysis

Baseline characteristics are expressed as the mean (±standard deviation) for normally distributed continuous variables, as median (interquartile range) for nonnormally distributed continuous variables, and as frequency (%) for categorical variables. Between-group differences were assessed by the *t*-test for normally distributed variables and by the Mann–Whitney *U* test for nonnormally distributed variables; proportions were compared using the *χ*^2^ test. Correlations were explored with Pearson's tests. Linear regression models were constructed for the multivariate analysis; results are expressed as beta with corresponding 95% confidence intervals (CI). A 2-tailed *p* < 0.05 was considered significant. Statistical analysis was carried out using SPSS Statistics version 23 (SPSS Inc, Chicago, USA).

## 3. Results

A total of 143 patients were assessed for eligibility; 26 patients were excluded (20 did not meet the inclusion criteria and 6 declined to participate), 117 patients after a recent myocardial infarction were included, and no participant had a previous history of arterial or venous thrombotic events. Baseline characteristics and risk factor presence are reported in Table 1. Medical therapy, prescribed at the time of the event, is listed in Table 1. The median triglyceride value was 115 mg/dL (IQR 88), median glucose value was 100 mg/dL (IQR 14), and median TyG index value was 8.68 (IQR 0.5). We divided participants according to the median value of the TyG index and compared the two groups: BMI and total cholesterol were found to be significantly increased and HDL cholesterol to be significantly decreased in the group with the TyG index above median (Tables 1 and 2). A comparison between the lowest and highest tertile of the TyG index was performed (Table 3); a significant difference was found in diabetes prevalence and a trend to significance for BMI. Among coagulation markers, OCP and OHP were found to be significantly higher in patients with the TyG index above median (Table 2).

A correlation analysis using Pearson's test showed a correlation between TyG index and total cholesterol (*r* = 0.382, *p* < 0.001), TyG index and HDL cholesterol (*r* = −0.364, *p* < 0.001), TyG index and LDL cholesterol (*r* = 0.199, *p* = 0.038), TyG index and OCP (*r* = 0.229, *p* = 0.026), TyG index and OHP (*r* = 0.202, *p* = 0.049), and TyG index and fibrinogen (*r* = 0.271, *p* = 0.005). No correlation was found between TyG index and OFP (*r* = −0.035, *p* = 0.738), TyG index and D-dimer (*r* = 0.077, *p* = 0.435), and TyG index and von Willebrand factor (*r* = 0.019, *p* = 0.846).

**Figure 1:**
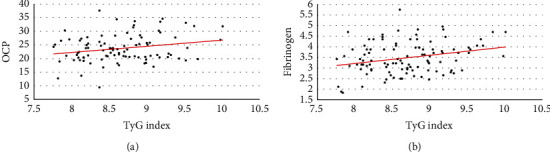
(a) Correlation between triglyceride-glucose index and overall coagulation potential; (b) correlation between triglyceride-glucose index and fibrinogen. TyG: triglyceride-glucose index; OCP: overall coagulation potential.

In the multivariate model which accounted for sex, age, and BMI, the correlation between TyG index and total cholesterol (*R*^2^ 0.184; ANOVA for regression *p* < 0.001; beta 0.362 [0.117-0.329], *p* < 0.001), between TyG index and HDL cholesterol (*R*^2^ 0.146; ANOVA for regression *p* < 0.001; beta -0.357 [-0.878–-0.238], *p* = 0.001), between TyG index and OCP (*R*^2^ 0.108; ANOVA for regression *p* = 0.035; beta 2.08 [0.79-4.01], *p* = 0.042), and between TyG index and fibrinogen (*R*^2^ 0.11; ANOVA for regression *p* = 0.015; beta 0.35 [0.08-0.62], *p* = 0.012) emerged as statistically significant (Figure 1).

To further explore the association between coagulation biomarkers and TyG index, a ROC analysis was performed. Both OCP and fibrinogen showed a modest predictivity for the TyG index (ROC area 0.60, 95% CI 0.480–0.713 and ROC area 0.59, 95% CI 0.484–0.701, respectively).

## 4. Discussion

In the present study, we have shown a significant association between the TyG index (a biomarker of cardiometabolic risk) and haemostatic abnormalities in patients after myocardial infarction. The finding suggests a possible link between cardiometabolic and coagulation derangements beyond traditional clinical features of the metabolic syndrome. To the best of our knowledge, ours is the first study to appraise a strong positive correlation between a procoagulant state (OCP and fibrinogen levels) and the TyG index, suggesting a pathophysiological interplay between cardiometabolic abnormalities and atherothrombotic potential in patients after a recent coronary event.

The metabolic syndrome is a known predictor of cardiovascular events in patients with established CAD [28–30]. Recent research has identified the TyG index as a reliable marker of metabolic syndrome and a strong predictor of CAD, premature CAD, and adverse events in CAD patients [10–13, 28]. In our study, values of the TyG index were comparable to those from previous studies in patients with CAD, wherein TyG index values were also associated with an increased event risk [11]. Our data complete these observations by proposing a pathophysiological link between the TyG index and haemostatic derangements; as the majority of CAD events represent thrombotic complications of atherosclerosis, an association between cardiometabolic and haemostatic derangements provides an important further insight in the unfavourable atherothrombotic prognostic role of the metabolic syndrome.

Previous studies have shown that insulin resistance and metabolic syndrome may confer a procoagulant state through several mechanisms. Adipose tissue may impair fibrinolysis through direct production of coagulation factors (such as PAI-1 and thrombin activatable fibrinolysis inhibition), dysmetabolism-associated nonalcohol liver steatosis may alter the hepatic synthesis of haemostatic factors, and adipokines may affect platelet function; insulin resistance-associated low-grade inflammation and endothelial dysfunction may modulate the genetic expression, activity, and interactions of haemostatic factors [27, 31–33]. In our study, we detected a strong correlation between known markers of metabolic disorder and TyG index and the association of the TyG index with a procoagulant state. The traditional assessment of a procoagulant state relies on measuring individual coagulant factors but may fail to capture the complexity of the overall haemostatic processes, and even in our study, D-dimer and von Willebrand factor did not show a significant correlation with the TyG index, but a strong correlation, independent of BMI, was found for fibrinogen, a known marker of coagulation. The finding supports coagulation activation in patients with metabolic syndrome and CAD. Global haemostatic assays for net yields of coagulation and fibrinolysis have recently been proposed as more suitable indicators for overall haemostasis appraisal [19]. They have been extensively validated in diverse populations, including patients with CAD [20, 24, 25, 34]. In our study, OCP levels—indicating hypercoagulability—were comparable to other studies of patients with CAD [23, 25] and significantly increased in patients with an elevated TyG index. Hence, our results corroborate that CAD confers a procoagulant state, which is especially pronounced in the CAD subpopulation with metabolic syndrome. Moreover, the association between TyG index and procoagulation was found to be independent of BMI. While clinical features of the metabolic syndrome (i.e., obesity, blood pressure, and metabolic dyslipidaemia) represent pivotal characteristics for the diagnosis and management of the condition, preclinical cardiometabolic biomarkers—such as the TyG index—may provide an independent, additional tool for early and meaningful detection of dysmetabolic derangements.

Hence, our results suggest that the TyG index is an independent marker of both cardiometabolic and haemostatic derangements. In this respect, an independent association of the TyG index and a procoagulant state in patients after a recent myocardial infarction may confer an important starting point for future risk assessment strategies for patients with CAD.

Limitations of our study primarily pertain to its realisation in a single centre and to its cross-sectional observational design and the study of surrogate biomarkers. Firstly, the single-centre nature of our study is somewhat counterbalanced by the fact that it was carried out at a national referral centre gathering a wide and varied patient population. In fact, the characteristics and risk factor presence in our participant group are similar to data from the literature, and hence, our results can be fairly generalized to the population of patients with CAD after a recent myocardial infarction. Secondly, the cross-sectional design can carry some measurement bias. Our measurements were in line with those reported in the literature for participants with CAD. Thirdly, we were able to include in the analysis only BMI as a marker of excess body fat. Including waist circumference or ectopic fat deposition would have added a deeper layer of understanding about the interplay between various components of the metabolic syndrome and coagulation markers. Fourthly, ours was a study to find an association; while it provides interesting and potentially important insights into the pathophysiological interplay between cardiometabolism and haemostasis in CAD, further prospective cohort studies are needed to establish a potential clinical relevance of our findings.

## 5. Conclusions

In conclusion, the TyG index represents an important and potentially relevant pathophysiological link between cardiometabolic and haemostatic derangements in patients with CAD. The TyG index is a reliable marker of metabolic syndrome and has a strong correlation with a procoagulant state. Our findings suggest that metabolic syndrome may be an important driver of atherothrombotic risk in patients with CAD.

## Figures and Tables

**Table 1:** Baseline demographic and risk factors.

	All (*n* = 117)	TyG below median (*n* = 58)	TyG above median (*n* = 59)	*p* value
Women^∗^	23 (19.7)	12 (20.7)	11 (18.9)	0.808
Age (years)^∗∗^	56 (10.3)	57.6 (10.8)	55.1 (10.0)	0.223
BMI (kg/m^2^)^∗∗^	28.8 (4.6)	28.1 (4.0)	29.9 (4.8)	0.013
Event				0.554
STEMI^∗^	71 (60.7)	35 (60.3)	36 (61.0)	
NSTE-ACS^∗^	46 (39.3)	23 (39.7)	23 (39.0)	
Arterial hypertension^∗^	86 (73.5)	44 (75.8)	42 (71.2)	0.495
Diabetes mellitus^∗^	10 (8.5)	4 (6.9)	6 (10.2)	0.093
Dyslipidaemia^∗^	75 (64.1)	37 (63.8)	38 (64.4)	0.423
Family history^∗^	46 (39.3)	24 (41.4)	22 (37.3)	0.695
Smoker^∗^	54 (46.2)	25 (43.1)	29 (49.2)	0.181
Antiplatelet drugs				0.940 0.824
ASA^∗^	116 (99.1)	58 (100)	58 (98.3)	
Clopidogrel^∗^	12 (10.3)	7 (12.1)	5 (8.5)	
Prasugrel^∗^	25 (21.4)	12 (20.7)	13 (22.0)	
Ticagrelor^∗^	73 (62.4)	36 (62.1)	37 (62.7)	
ACE inhibitor^∗^	91 (77.8)	47 (81.0)	44 (74.6)	0.615
Beta-blocker^∗^	95 (81.2)	46 (79.3)	49 (83.1)	0.580
Statins^∗^	117 (100)	58 (100)	59 (100)	0.761
Rosuvastatin^∗^	104 (88.9)	53 (91.4)	51 (86.4)	
Atorvastatin^∗^	13 (11.1)	5 (8.6)	8 (13.6)	

ASA: acetylsalicylic acid; BMI: body mass index; NSTE-ACS: non-ST-elevation acute coronary syndrome; SD: standard deviation; STEMI: ST-elevation myocardial infarction; TyG: triglyceride-glucose index; ∗: results displayed as number (%); ∗∗: results displayed as mean (standard deviation).

**Table 2:** Clinical, biochemical, and coagulation markers.

	All (*n* = 117)	TyG below median (*n* = 58)	TyG above median (*n* = 59)	*p* value
Systolic blood pressure (mmHg)^∗^	124.1 (7.1)	122.8 (15.8)	125.7 (17.6)	0.387^a^
Diastolic blood pressure (mmHg)^∗^	78.8 (10.3)	77.4 (10.7)	80.5 (10.2)	0.132^a^
Total cholesterol (mg/dL)^∗∗^	123.7 (108.3, 150.8)	119.9 (104.4, 143.1)	127.6 (112.1, 158.6)	0.045^b^
HDL cholesterol (mg/dL)^∗∗^	42.5 (34.8, 50.3)	42.5 (38.7, 54.1)	38.7 (34.8, 46.4)	<0.001^b^
LDL cholesterol (mg/dL)^∗∗^	58 (42.5, 73.5)	54.1 (46.4, 69.6)	58 (42.5, 81.2)	0.617^b^
OCP, abs-sum^∗∗^	22.6 (20.4, 26.6)	22.4 (20.3, 25.5)	24.3 (20.6, 29.2)	0.040^b^
OHP, abs-sum^∗∗^	8.0 (6.5, 9.8)	7.5 (5.6, 9.1)	8.3 (6.9, 9.8)	0.041^b^
OFP (%)^∗∗^	66.0 (60.0, 71.0)	66.0 (60, 73)	66 (60.3, 69)	0.517^b^
Fibrinogen (g/L)^∗∗^	3.3 (2.9, 3.9)	3.2 (2.9, 3.9)	3.6 (3.0, 4.0)	0.092^b^
D-dimer (*μ*g/L)^∗∗^	304.5 (197.0, 478.0)	293.0 (215.5, 404.5)	333.0 (195.0, 572.5)	0.414^b^
von Willebrand factor (%)^∗∗^	150.5 (120.5, 192.5)	141.0 (115.0, 180.0)	169.0 (124.5, 205.0)	0.146^b^
Time from AMI to visit (days)^∗∗^	58.0 (47.5, 79.5)	58.0 (49.0, 82.0)	56.0 (47.0, 79.0)	0.959^b^

AMI: acute myocardial infarction; OCP: overall coagulation potential; OFP: overall fibrinolytic potential; OHP: overall haemostatic potential; TyG: triglyceride-glucose index; ∗: results displayed as mean (SD); ∗∗: results displayed as median (25^th^, 75^th^ percentile). ^a^*t*-test; ^b^Mann–Whitney *U* test.

**Table 3:** Baseline demographic and risk factor comparison between lowest and highest tertile of TyG index.

	Lowest tertile TyG index (*n* = 37)	Highest tertile TyG index (*n* = 36)	*p* value
Women^∗^	9 (24)	8 (22)	0.309
Age (year)^∗∗^	57.2 (10.7)	54.9 (9.9)	0.339
BMI (kg/m^2^)^∗∗^	28.1 (4.5)	30.4 (5.5)	0.057
Event			0.665
STEMI^∗^	21 (56.8)	24 (66.7)	
NSTE-ACS^∗^	16 (43.2)	12 (33.3)	
Arterial hypertension^∗^	29 (78.4)	27 (75.0)	0.936
Diabetes mellitus^∗^	1 (2.7)	8 (22.2)	0.004
Dyslipidaemia^∗^	23 (62.2)	25 (69.4)	0.793
Family history^∗^	17 (45.9)	42 (38.5)	0.401
Smoker^∗^	15 (40.5)	19 (52.8)	0.564

BMI: body mass index; NSTE-ACS: non-ST-elevation acute coronary syndrome; SD: standard deviation; STEMI: ST-elevation myocardial infarction; TyG: triglyceride-glucose index; ∗: results displayed as number (%); ∗∗: results displayed as mean (standard deviation).

## Data Availability

The data used to support the findings of this study are available upon request to the corresponding author.
